# Nursing teamwork is essential in promoting patient-centered care: a cross-sectional study

**DOI:** 10.1186/s12912-023-01592-3

**Published:** 2023-11-17

**Authors:** Hyang Baek, Kihye Han, Hyeonmi Cho, Jieun Ju

**Affiliations:** 1 School of Nursing, University of Maryland, Baltimore, MD 21201 USA; 2 College of Nursing, Chung-Ang University, Seoul, 06974 South Korea; 3 Mo-Im Kim Nursing Research Institute, College of Nursing, Yonsei University, Seoul, 03722 South Korea; 4 Graduate School, Chung-Ang University, Seoul, 06974 South Korea

**Keywords:** Latent profile analysis, Nurse staffing, Nursing teamwork, Patient-centered care, Work environment

## Abstract

**Background:**

There has been little research regarding nursing teamwork, despite its important role in multidisciplinary teamwork in healthcare settings and its significance in ensuring high-quality nursing care. This study aimed to determine the teamwork levels of Korean nurses and examine the relationship between nursing teamwork and patient-centered care while controlling for other individual and work-related factors.

**Methods:**

We conducted a cross-sectional analysis of online survey data. The study population consisted of 992 Korean registered nurses employed in hospitals who had a minimum of six months of clinical experience. We performed latent profile analysis to identify latent teamwork subgroups based on response patterns. We performed analysis of variance and Chi-square tests to examine differences in individual and work-related characteristics according to teamwork group. We used multiple linear regression to investigate how nursing teamwork could affect patient-centered care after controlling for covariates.

**Results:**

We identified three nursing teamwork subgroups: low, mid, and high. Nurses with a higher level of teamwork in their units tended to work fewer hours with more adequate staffing (*F* = 5.88, *p* = 0.003 for working hours; *F* = 7.68, *p* < 0.001 for staffing adequacy). There was a significant positive association between nursing teamwork and patient-centered care after controlling for personal and work-related characteristics. Compared with low teamwork, mid and high teamwork increased patient-centered care scores by 0.32 (95% confidence interval [CI] = 0.23–0.40) and 0.57 (95% CI = 0.48–0.66), respectively.

**Conclusion:**

Our findings indicate that enhancing nursing teamwork can serve as an effective strategy for promoting patient-centered care. Providing nurse education and training to equip nurses with the necessary knowledge and skills for effective teamwork is a crucial step. Additionally, fostering management commitment to create a supportive working environment, including adequate staffing, can facilitate improved nursing teamwork and, subsequently, patient-centered care.

## Background

Patient-centered care (PCC), often used interchangeably with person-focused or patient-focused care [1], is the standard for nursing care [2]. PCC is an approach that puts the individual patient at the center of the care process and consists of three components: holistic, collaborative, and responsive care [3]. This process can be implemented independently or collaboratively between healthcare professionals and patients to reflect patient needs and preferences [4]. It is highly valued as a critical component of healthcare reform in the United States [5] and is beneficial to patients, healthcare providers, and organizations [6]. Previous studies have indicated that PCC is associated with less hospitalization and fewer laboratory and diagnostic tests, leading to medical cost reduction [7, 8], improved staff productivity, and better resource allocation [6]. Furthermore, patient participation in the care process allows them to maintain control over their own lives and improves their quality of life [9]. For nurses, PCC has been found to correlate with their well-being and job satisfaction [10]. To motivate nurses to actively engage in providing PCC, potential factors that can influence the delivery of PCC need to be investigated.

In recent years, multidisciplinary teamwork, referring to teamwork among physicians, nurses, and other relevant healthcare professionals in providing patient care [11], has attracted attention in healthcare research. Previous studies have shown that multidisciplinary teamwork leads to better PCC [12]. Teamwork failures cause more than 70% of sentinel events in the healthcare system [13, 14]. As nurses constitute a significant portion of the global healthcare workforce, fostering stronger teamwork among nursing staff is essential to promote effective multidisciplinary teamwork [15]. Therefore, it is imperative to further investigate the dynamics and implications of nursing teamwork [16]. Nurses are frontline care providers who spend the most time with patients, constantly monitoring and supporting them. Effective nursing teamwork, defined as effective work between two or more nurses who work together in the same unit to provide care and perform other tasks for patients [14], is vital for the provision of high-quality healthcare [17]. Nurses serve as the foundation for multidisciplinary teamwork in healthcare settings as intermediaries between various healthcare providers [18]. Therefore, determining whether and how nursing teamwork could contribute to PCC may provide guidelines for its facilitation.

The composition of nursing teams can vary substantially by country. In the United States, a nursing team usually consists of registered nurses (RNs), licensed practical nurses (LPNs), nursing assistants (NAs), and unit secretaries (USs) in the same unit in a hospital. In Korean hospitals, RNs and NAs working closely together in one unit typically comprise a nursing team. According to Kaiser and Westers [18], nursing team members have a common goal and the same conceptualization of what to do and how to do it. They understand their own responsibilities and each other’s strengths and weaknesses [18]. They continuously monitor each other, communicate, share information and knowledge, and provide help willingly when other team members are in need, which enhances the continuity of care [18]. In such a collaborative team environment, not only are nurses heard and respected, but patients are also more likely to be engaged in their care.

While the need for the further investigation of nursing teamwork and PCC persists, a deeper understanding of this relationship requires the consideration of other organizational factors, as PCC approaches involve all levels of an organization [19, 20]. For example, while a staffing shortage is a barrier to effective care [21–23], previous research implies that effective teamwork can improve nursing care quality when staffing levels are adequate [24, 25]. Without adequate levels of teamwork and staffing, the core factors of PCC, such as patient education and communication with or comfort for patients and families, can be missed [25]. The importance of nursing teamwork in PCC remains an understudied area. To address this research gap, our study aimed to investigate the relationship between nursing teamwork and PCC.

## Methods

### Aims

This study aimed to [1] determine the level of teamwork among Korean hospital nurses and [2] investigate how nursing teamwork affects PCC in hospital settings, while statistically controlling for other individual- and work-related factors through quantitative analysis. We attempted to generalize the association between nursing teamwork and PCC using a sample of nurses from Korea [26]. We hypothesized that enhanced nursing teamwork may positively affect PCC.

### Study design and sampling procedure

This study employed a cross-sectional design using online survey data. It was part of a research project on nursing teamwork related to nursing and patient outcomes. Data were collected from Korean nurses in May 2021. The data collection process was described in a previous study [27]. In brief, study participants were recruited from ‘My Duty,’ a work scheduler mobile application designed for shift-work nurses used by approximately 85% of Korean nurses. Current users were invited to participate in the survey via the mobile application and could complete it after they provided consent. We included RNs working in hospital settings with 100 beds or more who had over six months of clinical experience. For this study, we excluded nurses who were as the only nursing personnel in their units because nursing teamwork requires at least two nurses in the unit.

### Measures

Nurses were asked about nursing teamwork within their units (as an independent variable) and PCC (as a dependent variable). Additionally, we measured work-related and individual characteristics as confounding variables.

#### Nursing teamwork

Nursing teamwork was measured using the Korean version of the Nursing Teamwork Survey (NTS-K) [14, 27]. We defined a nursing team as nurses and NAs working together in the same unit. It consists of 33 items in five sub-domains: trust (7 items), team orientation (9 items), backup (6 items), shared mental model (7 items), and team leadership (4 items), and is one of the most widely used instruments for measuring nursing teamwork levels. Previous studies have reported its strong validity – content, concurrent, construct [14, 27] – and good reliability (Cronbach’s alpha = 0.825). The items were rated on a 5-point Likert scale (1 = rarely to 5 = always). The mean score of each sub-domain was calculated. We performed latent profile analysis (LPA) using the five sub-domain scores. LPA is a statistical modeling approach used to identify latent subgroups within a population based on continuous variables, presenting group membership as a categorical variable [28]. This could capture the heterogeneity of individual perceptions of nursing teamwork instead of creating a variable using the overall mean values. In this study, the three groups obtained by LPA indicated the three different levels of perceived nursing teamwork (low, medium, or high). We coded the three levels as a variable and used it as an independent variable representing the degree of nursing teamwork.

#### Patient-centered care (PCC)

For this study, we used the Korean version of the PCC scale [4, 29]. It consists of 23 items, including 8 items for holistic care, 9 items for collaborative care, and 6 items for responsive care. Each item was rated on a 5-point Likert scale (1 = strongly disagree to 5 = strongly agree). We used the mean score of the 23 items as the overall score, with a higher score indicating a higher level of PCC provision as perceived by nurses. The measure had acceptable content and construct validity [4, 29], and its internal consistency was good (Cronbach’s alpha = 0.935 in Lee et al. [29]; 0.949 in this study).

#### Work-related and individual characteristics

Nurses were asked about their work-related characteristics, including working hours per week, years of work experience in the current unit, unit type, and hospital size. Nurses rated the perceived staffing adequacy during the last month using a 5-point Likert scale (1 = rarely, 2 = 25% of the time, 3 = 50% of the time, 4 = 75% of the time, 5 = always). We included age as a covariate based on previous research findings [24].

### Statistical analysis

We used SPSS 27 and Mplus 8.6 for statistical analysis. Our sample size of 992 was considered sufficient for both LPA and multiple linear regression [30, 31]. After screening and cleaning the data using SPSS, we identified latent clusters based on the five NTS sub-domain scores by LPA with Mplus software. To determine the number of subgroups, we generated and compared several model fit indices, including Akaike information criterion (AIC), Bayesian information criterion (BIC), sample-size adjusted BIC, entropy, Lo-Mendell-Rubin adjusted likelihood ratio, and bootstrapped likelihood ratio test statistics [32]. We used membership in different teamwork groups as a categorical predictor for further analysis. We performed an analysis of variance (ANOVA) and the Chi-square test to examine the differences in individual and work-related characteristics according to the teamwork group. We used multiple linear regression to investigate whether the level of nursing teamwork could predict PCC while controlling for other work-related characteristics (working hours per week, work experience in the current unit, staffing adequacy, unit type, and hospital size) and age as a demographic variable. We examined assumptions for multiple linear regression (normality, linearity, multicollinearity, homoscedasticity, and independence of residuals), which revealed no violation.

## Results

### Sample description

**Table 1:** Characteristics of Korean hospital nurses according to the nursing teamwork level, n = 992

	Overall	Nursing teamwork level			Test for difference	
		low n = 197	mid n = 501	high n = 294	* F or χ²*	*p*
	*M (SD)*	*M (SD)*	*M (SD)*	*M (SD)*		
Age (years)	29.0 (4.6)	29.0 (4.6)	28.9 (4.9)	29.1 (5.1)	0.20^a^	0.822
Working hours per week	44.8 (14.2)	47.5 (13.6)	44.7 (13.8)	43.1 (14.8)	5.88^a^	0.003
Years working in the unit	3.5 (3.2)	3.2 (2.5)	3.5 (3.2)	3.7 (3.6)	1.30^a^	0.274
Staffing adequacy	2.8 (1.2)	2.6 (1.2)	2.8 (1.2)	3.0 (1.3)	7.68^a^	< 0.001
Patient-centered care	3.6 (0.5)	3.3 (0.6)	3.6 (0.5)	3.9 (0.5)	74.64^a^	< 0.001
	%	%	%	%		
Unit type						
General wards	72.3	76.1	72.3	69.7	4.18^b^	0.382
ICU/ER	25.0	20.3	25.5	27.2		
Others	2.7	3.6	2.2	3.1		
Hospital size (beds)						
100–399	27.9	33.0	25.0	29.6	12.66^b^	0.049
400–699	17.8	17.3	18.4	17.3		
700–999	24.8	25.4	27.7	19.4		
> 1000	29.4	24.4	28.9	33.7		

*Note.* M = mean; SD = standard deviation; ^a^ = analysis of variance; ^b^ = Chi-square test; ICU = intensive care unit; ER = emergency room

Of the 1,160 participants who completed responses, 45 were disqualified for various reasons during data cleaning (e.g., not a nurse or clinical experience of less than six months). We excluded another 123 participants in some nursing units (OR/PACU, outpatient, and NICU) because the ‘PCC’ and ‘teamwork’ measures were not validated or applicable. Finally, a total of 992 nurses were included in this analysis.

On average, the sampled nurses were 29 years of age (SD = 4.6) and had 3.5 years of working experience (SD = 3.2) in their current units (Table 1). They reported a mean of 44.8 working hours per week, including overtime, during the last month. Over 90% of the nurses worked in general hospitals and did shift work (two or three shifts). Most nurses (72%) worked in general wards (e.g., medical/surgical units), with 25% in intensive care units (ICU) or emergency departments (ER).

### Nursing teamwork profile

**Table 2:** LPA indices for determining the group number

**Number of classes**	**Log-likelihood**	**Number of free parameters**	**AIC**	**BIC**	**Sample-size adjusted BIC**	**Entropy**	**LMR LRT test**		**Bootstrap LRT**	**Number of participants in each class**	
							value	*p*	*p*		
2	-4247.14	16	8526.29	8604.68	8553.87	0.85	1735.14	< 0.001	< 0.001	361/631	
3	-3879.89	22	7803.78	7911.58	7841.71	0.851	**717.18**	**< 0.001**	< 0.001	197/501/294	
4	-3767.20	28	7590.41	7727.60	7638.67	0.83	**220.06**	**0.446**	< 0.001	224/85/441/242	

*Note.* AIC = Akaike information criterion; BIC = Bayesian information criterion; LMR LRT test = Lo-Mendell-Rubin adjusted likelihood ratio test

**Fig. 1:**
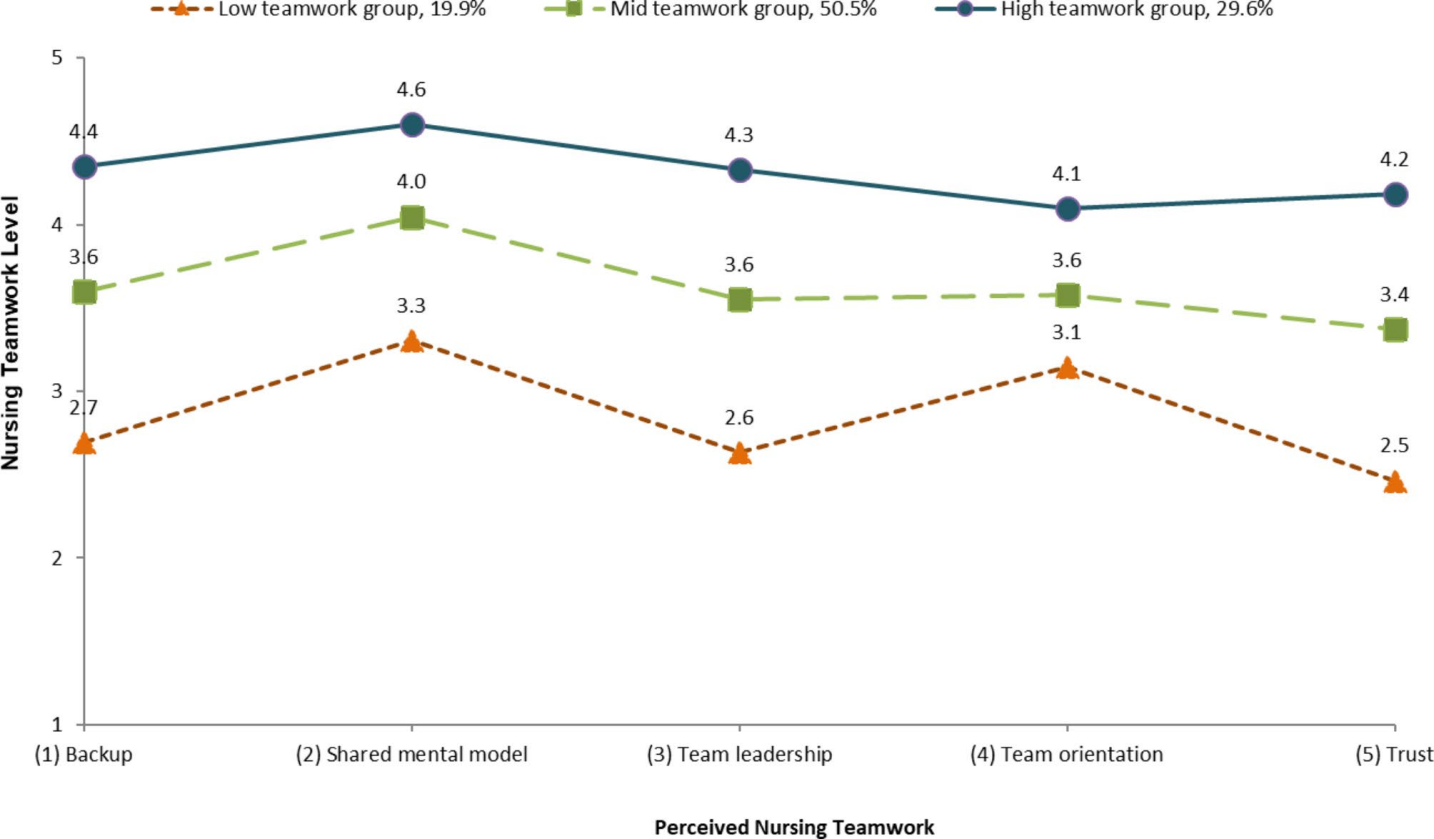
Perceived nursing teamwork levels of three teamwork groups Note: The three groups were determined by LPA based on their responses to the five nursing teamwork sub-domains. The items were rated on a 5-point Likert scale (1 = rarely to 5 = always)

Several model fit indices were compared between the classes (Table 2). The model utilizing the three subgroups demonstrated the best fit (Table 2) based on the Lo-Mendell-Rubin adjusted likelihood ratio (p < 0.001) in the three-group analysis; however, the result was not statistically significant in the four-group analysis. The subgroups were classified as low, mid, and high teamwork based on the observed characteristics (Fig. 1). Approximately 20% of the nurses were included in the low teamwork group, 50% in the mid teamwork group, and 30% in the high teamwork group. The mean scores of all five NTS sub-domains in the high teamwork group were higher than those in the other two groups. The low teamwork group had the lowest scores for all of the five sub-domains.

### Individual and work-related characteristics according to the nursing teamwork level

There were significant differences in weekly work hours and staffing adequacy according to the perceived nursing teamwork level (Table 1). Nurses who perceived a higher level of teamwork in their units tended to work fewer hours with more adequate staffing (*F* = 5.88, *p* = 0.003 for working hours; *F* = 7.68, *p* < 0.001 for staffing adequacy). No significant differences were noted in age, work experience in the current unit, unit type, or hospital size between the teamwork subgroups. Participants with a higher nursing teamwork level had a higher level of PCC (*M* = 3.3 for low teamwork, 3.6 for mid teamwork, 3.9 for high teamwork; *F* = 74.64, *p* < 0.001).

### Nursing teamwork profile and PCC

**Table 3:** Coefficients of predictors for patient-centered care among Korean hospital nurses, n = 992

	Patient-centered care			
	*B*	*p*	*95% CI*	
			*Lower*	*Upper*
Constant	3.41	< 0.001	3.15	3.67
Nursing teamwork = mid (reference = low)	0.32	< 0.001	0.23	0.40
= high (reference = low)	0.57	< 0.001	0.48	0.66
Staffing adequacy	0.01	0.521	-0.02	0.03
Working hours per week	0.00	0.812	0.00	0.00
Unit = ICU/ER (reference = general wards)	-0.03	0.493	-0.10	0.05
= others (reference = general wards)	-0.09	0.347	-0.29	0.10
Hospital size = 400–699 (reference = 100–399)	-0.07	0.188	-0.16	0.03
= 700–999 (reference = 100–399)	-0.01	0.908	-0.09	0.08
≥ 1,000 (reference = 100–399)	-0.03	0.541	-0.11	0.06
Age (years)	0.00	0.449	-0.01	0.01
Years working in the unit	-0.01	0.161	-0.02	0.00

*Note.* The coefficients were generated from multiple linear regression. CI = confidence interval; ICU = intensive care unit; ER = emergency room

Table 3 shows the association between nursing teamwork and PCC while controlling for individual and work-related characteristics. The model containing all predictors (nursing teamwork, working hours, work experience, staffing adequacy, unit type, hospital size, and age) was significant (*F* = 15.738, *df* = 10, 981, *p* < 0.001), explaining 13.8% of the variance in PCC (adjusted R^2^ = 0.129). The PCC scores increased by 0.32 (95% CI = 0.23–0.40) and 0.57 (95% CI = 0.48–0.66) with mid and high levels of teamwork, respectively, compared to low teamwork.

## Discussion

PCC provision is challenging due to its complexity. Therefore, compared with task-oriented care, it often requires more time for nurses to understand the patient’s preferences and values and to reflect and coordinate them in the care process [33]. PCC can be affected by various factors, including individual nurse characteristics, such as age, compassion satisfaction, and burnout status [34]. These factors may not be amenable or could burden nurses if changes are forced. Institutional and organizational efforts could be more effective in creating a work environment to support PCC. Research on PCC indicates that a lack of time could lead to a discrepancy between what nurses know and what they actually do for PCC [33]. Currently, nursing techniques may not be profitable for hospitals, as their costs are usually included in procedures. Hospitals may be reluctant to prioritize investment in a favorable environment for quality nursing. Nevertheless, PCC is associated with positive patient and nursing outcomes [11, 35, 36], which will ultimately reduce operational costs and human resource requirements for hospitals. Our results showed that improving nursing teamwork could be a strategy for increasing PCC. Further training programs for nurses to empower them with the necessary knowledge and skills are recommended [34].

We identified three subgroups within the study sample based on the response pattern of NTS sub-domains. Each subgroup was defined according to the levels of NTS sub-domains (high teamwork group, mid teamwork group, and low teamwork group). In this study, the five NTS sub-domains were classified based on levels rather than distinct characteristics, which indicated that the components of nursing teamwork are not clearly distinguishable. Rather, they are interrelated concepts that change collectively. Indeed, the Salas theory, which is a framework for the NTS, assumes that interrelationships among the components of teamwork are facilitated by coordinating mechanisms [14, 37]. A previous study examining the facets of nursing teamwork in multiple acute care environments observed a constant trend in NTS sub-domains regardless of the nursing unit type [23], which is consistent with our findings. In addition, various studies have reported that NTS sub-domains have a moderate-to-strong correlation with each other [14, 38].

In this study, a higher teamwork level was significantly associated with PCC. Similarly, Kuosmannen et al. [9] performed an integrative review and found that interdisciplinary teamwork and an environment conducive to open communication are prerequisites for PCC. PCC emphasizes information exchange and shared decision-making between patients, families, and healthcare providers [6, 39], which could explain the close relationship between nursing teamwork and PCC. When nurses within a team can share opinions based on their expertise and work together on nursing care plans, care is well coordinated and patient-focused. Furthermore, a culture of respect and open communication among nurses could influence communication with patients accordingly. Limited studies have examined nursing teamwork in relation to PCC. Most previous studies have focused on interdisciplinary teamwork and nurses’ collaboration with other healthcare workers. With a person-focused approach using LPA, this study showed a significant association between nursing teamwork and PCC. Therefore, organizational strategies should be implemented to support teamwork in nursing. More research is warranted to elucidate the nursing teamwork concept.

Nursing teamwork was significantly associated with work-related factors. Nurses with a lower level of nursing teamwork worked in a more austere environment. Longer working hours and inappropriate staffing levels could lead to heavier workloads [40–42]. This may deprive nurses of the energy or time to monitor and support each other and exchange information, all of which are required for effective teamwork [23, 36, 37]. Similar to previous studies, the results demonstrated that staffing adequacy may be closely related to overall nursing teamwork [24, 41, 43]. Adequate staffing allows team members to flexibly react to team tasks, thus improving team functioning [44]. Nursing teamwork is an effective strategy for preventing missed care [45] and increasing job satisfaction [46]. However, a lack of resources or support could hinder the creation of a supportive environment that promotes nursing teamwork.

While our research provides valuable insights, several limitations warrant consideration for a comprehensive understanding of the study results. The adjusted R-squared of our model is small, despite the significance. Our main objective was to investigate the influence of nursing teamwork on patient-centered care, rather than to predict the behavior of patient-centered care delivery. Notably, we observed a statistically significant B value for nursing teamwork concerning patient-centered care, rendering the acceptable R-squared value [47]. Our research findings were based on a cross-sectional dataset; thus, we cannot guarantee causal relationships among variables. Future research with a longitudinal design is warranted to examine the causal relationship between nursing teamwork and PCC.

This study was based on self-reported measures. In particular, it measured nurses’ perceptions of PCC, which could differ significantly from their ability or availability to provide PCC. There is a lack of research on the actual provision of PCC [48]. Repetitive studies with objective measures or studies including patient perspectives would be valuable. This study used convenience sampling by recruiting participants through a mobile application, possibly causing selection bias due to technology accessibility. We limited our sample of nurses to RNs who worked in hospitals with > 100 beds and who performed shift work, while the original nursing teamwork study [16] measured teamwork between RNs, LPNs, NAs, and USs. This may limit our study’s generalizability [49]. Future studies should include random sampling with a larger number of participants from a variety of settings and positions.

## Conclusion

PCC requires active collaboration and respectful conversation between healthcare professionals and patients, which contributes to an interactive healthcare environment [19]. In this context, this study highlights the importance of nursing teamwork for PCC. To improve nursing teamwork in clinical practice, management commitment and relevant policies should be prioritized. In addition, managerial efforts to ensure a supportive working environment, such as optimal staffing and maintaining proper working hours or schedules, support strong nursing teamwork, which in turn could enhance nurses’ PCC practice. Education through awareness and knowledge starting from the undergraduate level (e.g., TeamSTEPPS) will provide basic skills for better teamwork [50], as well as organizational training such as virtual simulation and train-the-trainer interventions aimed at improving teamwork skills [51, 52], would be helpful for PCC. Further research is recommended on effective teamwork and its effect on PCC.

## Data Availability

The data that support the findings of this study are available from the corresponding author upon reasonable request.
